# Temporal Variation of Earthworm Impacts on Soil Organic Carbon under Different Tillage Systems

**DOI:** 10.3390/ijerph16111908

**Published:** 2019-05-30

**Authors:** Yafei Guo, Xiaoping Zhang, Yan Zhang, Donghui Wu, Neil McLaughlin, Shixiu Zhang, Xuewen Chen, Shuxia Jia, Aizhen Liang

**Affiliations:** 1 Key Laboratory of Mollisols Agroecology, Northeast Institute of Geography and Agroecology, Chinese Academy of Sciences, Changchun 130102, China; guoyafei15@mails.ucas.ac.cn (Y.G.); zhangxiaoping@neigae.ac.cn (X.Z.); zhangyan13@mails.ucas.ac.cn (Y.Z.); wudonghui@iga.ac.cn (D.W.); zhangshixiu@neigae.ac.cn (S.Z.); chenxuewen@neigae.ac.cn (X.C.); jiashuxia@neigae.ac.cn (S.J.); 2 Department of Resources and Environment, University of Chinese Academy of Sciences, Beijing 100049, China; 3 Ottawa Research and Development Centre, Agriculture and Agri-Food Canada, Ottawa, ON K1A0C6, Canada; neil.mclaughlin@sympatico.ca

**Keywords:** earthworm, residue return, carbon dynamics, conservation tillage, conventional tillage

## Abstract

Previous research has shown the varied effect of earthworms on soil carbon dynamics. We carried out a 180-day incubation experiment with earthworms and maize residue additions under conventional tillage (CT) and no tillage (NT) system conditions to quantify the earthworm effect in the black soil of northeastern China. Earthworms did not affect soil CO_2_ emissions, while residue addition significantly increased such emissions. The effects of earthworms on dissolved organic carbon (DOC) and microbial biomass carbon (MBC) gradually weakened with time in CT with and without residue addition, but gradually increased with time in NT with residue addition. In the CT system, earthworms accelerated the soil organic carbon (SOC) mineralization; and the newly added residue decomposed into SOC. In the NT system, earthworms accelerated the decomposition of native residues increasing the SOC content; this increase in decomposition rates by earthworms was greater than the inhibitory effect imposed by the addition of the new residue. Earthworms and residues combine to play a single role in CT and NT. This result will help in the understanding of the role of earthworms and residue in SOC dynamics, and in the development of management strategies to improve SOC.

## 1. Introduction

Dyson [1] showed the possibility of soil carbon (C) sequestration and, a lot of research has been done on the potential of, and prerequisites for, C sequestration in agricultural soils [2]. There are two normal ways to deal the residue in agricultural soils, namely keeping residues on the surface (conservation tillage) or removing residue for other purposes such as feeding livestock or as fuel (conventional tillage, the traditional farming practice in northeast China). Conservation tillage can enhance C protection and increase soil organic carbon (SOC), and can effectively increase soil nitrogen, and convert agricultural soils from C sources to C sinks by returning crop residues to the soil [2]. Under conservation tillage, returning residues can effectively increase SOC and nitrogen. Soil invertebrates like earthworms play an important function in soil processes at different spatial and temporal scales and also play an essential role in ecosystem services [3,4]. Fonte [5] and Blouin [4] found that earthworms are key actors of soil fertility in agricultural soils and are important regulators of soil structuring processes, organic matter dynamics and their integration in the soil. Earthworms function as keystone detritivores and ecosystem engineers [6,7,8] and play an integral role in the processes of soil formation and function [9] and the maintenance of the soil structure, and have a positive influence on physicochemical properties of soils [4,8,10]. As ecosystem engineers dwelling within the soil, they are capable of influencing soil carbon dynamics [9,11,12].

Some studies have shown that earthworms can increase the incorporation of residual C into soil aggregates in the short-term, and that earthworms aid in the decomposition of soil organic carbon (SOC) in the long-term [13]. The most important effects of earthworm activities on C cycling are by their feeding, burrowing and casting behavior [14]. Earthworms can promote C stabilization in macroaggregates and microaggregates formed in their casts [15,16,17]. Other short-term studies have reported that earthworms can increase carbon dioxide (CO_2_) emission from soils, thus suggesting increased decomposition in the longer term [13]. The reason is that earthworms can stimulate and accelerate organic matter (OM) decomposition by enhancing microbial respiration [18,19], and by fragmentizing, ingesting, disintegrating and transporting fresh plant material into the soil [20,21].

Scientists have different opinions about whether the earthworms increase or decrease SOC storage in the long term [22,23]. Hedde et al., [24] proposed that different agroecosystem management systems influence the magnitude and direction of the effect of earthworms on C dynamics. Hugh et al., [25] also showed that earthworm activity was lower, had lower density and lower biomass in reduced tillage compared to annual ploughing in an arable system without addition of organic materials in an 18 year experiment site. We still do not completely understand how earthworms and management practices interact, and their long-term function in agro-ecosystems [26]. Moreover, it is essential to study how earthworms interact with microbiota and thereby affect the C cycle [11,27,28].

The objectives of the present study were to identify the impact of earthworms on C dynamics in a 180-day mesocosm experiment with and without return of aboveground plant residue, and to investigate the difference between conventional tillage (CT) and no tillage (NT). We hypothesized that: (1) the effect of earthworms on C dynamics is different in CT and NT (with and without soil disturbance) and (2) the effect of earthworm on C dynamics is different when residue is or is not left on the soil surface.

## 2. Materials and Methods

### 2.1. Soil and Earthworm Collection

Soil samples were taken at Experimental Station (44°12′N, 125°33′E) of the Northeast Institute of Geography and Agroecology, Chinese Academy of Sciences, in Dehui County, Jilin Province, China. The field experiment with different tillage systems (CT and NT) was initiated in 2001. One crop a year and maize-soybean rotation system was applied in both CT and NT. Herbicides were used for weed control, but no insecticides were used after 2001. Tillage management for CT included removal of plant residue after harvest, fall moldboard ploughing, manually replacing residue after ploughing, spring cultivation, planting, and one or two post planting cultivations as required for weed control. There was no soil disturbance in NT except for planting; maize was manually harvested and the residue was manually cut into 30 to 35 cm lengths and left on the soil surface. Both CT and NT were planted using a no-till planter. The soil is a clay loam (Typic Hapludoll, USDA Soil Taxonomy) with an average of 36.0% clay, 24.5% silt, and 39.5% sand. The pH is 5.90 in CT and 5.87 in NT. The C: N ratio is 12.55 in CT and 12.05 in NT. Undisturbed soil samples were obtained from the maize phase of the tillage and rotation study site after harvest in October, 2016. We vertically inserted PVC pipe (10-cm diameter and 15-cm height) into the NT and CT soils to 15 cm depth and carefully removed the pipes with soil cores to avoid soil disturbance. Soil core samples were taken back to the lab for an incubation experiment.

### 2.2. Incubation Experiment

We had four different combinations of earthworm (E) and residue (S) treatments in each of CT and NT (ES, with earthworm and with residue addition; EN, with earthworm and without residue addition; NS, without earthworm and with residue addition; NN, without earthworm and without residue addition There were four replicates for each treatment.

We added 4.5 g of maize residue (a mixture of all of the above ground maize residue components), that was cut to about 4 mm length) to the surface of with residue treatments (NS and ES) in both NT and CT.

We added three mature earthworms (*Eisenia fetida*; 0.4 ± 0.16 g; middle age; the common species in this field) [29,30] to the surface of CT and NT soils with earthworm treatment, ES and EN.

Neither residue nor earthworms were added to the NN treatment in CT and NT.

After the residue and earthworms were added to the respective treatments, the bottoms of cores were wrapped with plastic film and the top was enclosed with nylon mesh to prevent earthworms from escaping, and the samples were allowed to sit at room temperature for 24 h. All core samples were then placed in an incubator (Memmert, HPP 750, Schwabach, Germany) with constant temperature of 18 °C (average temperature over the growing season of our study field site between 2005 and 2015) and air relative humidity of 50% to incubate in the dark.

A subset of four core samples for CT and four core samples for NT were oven-dried at 105 °C for 8 h to calculate the initial water content. We then calculated the weight of the samples needed to achieve 30% gravimetric water content. Each day, all samples in the incubator were weighed and water was added to adjust the gravimetric soil moisture to 30%.

### 2.3. Soil Sampling and Measurements in the Incubation Experiment

Four core samples were randomly selected from each treatment for respiration measurements. The samples were sealed, allowed to sit for three minutes, and respiration measurements made with a Licor-820 gas analyzer (LiCor-Biosciences, Lincoln, NE, USA). The respiration measurements were made on the same samples every day for the first 13 days, every 2 days for the next 16 days, every 3 days for the next 18 days, every 4 days for the next 12 days and finally every 7 days for the remainder of the 180 day measurement period; samples were returned to the incubator immediately after respiration measurements. The total CO_2_ emissions of our period were calculated by summing the total CO_2_ emissions of each day. The total CO_2_ emissions were calculated by numerical integration of the measured respiration rate data over the 180 day incubation period using the trapezoidal method.

We randomly selected four cores from each treatment at 30, 60, 120 and 180 days and destructively sampled the soil at 0-5 cm depth for SOC and active fraction C measurements. A sub-sample was oven-dried at 105 °C for 8 h to calculate water content. Visible plant residues and stones were removed and fresh soil subsamples were kept for dissolved organic carbon (DOC) and microbial biomass carbon (MBC) measurements.

A 10 g sample of fresh soil was put into a bottle with 50 mL K_2_SO_4_ (0.5 mol·L^−1^), and shaken for 1 hour at 20 °C and 200 revolutions min^-1^, allowed to rest at 0 °C for 1 hour, and then passed through a 0.45 μm filter. The DOC was measured using a TOC analyser (Multi C/N 3000, Analytik Jena, Jena, Germany). The MBC was determined using the fumigation-extraction method [31]; the extracted solutions of MBC were measured using the same TOC analyser as for DOC. MBC was calculated as Ec/K_EC_, where Ec = (organic C extracted from fumigated soil)-(organic C extracted from non-fumigated soil) and K_EC_ = 0.38 [32].

Soil samples for SOC measurement were gently broken, air-dried, and passed through a 0.154 mm sieve. The total carbon of soil was determined using a Flash EA 1112 elemental analyser (Thermo-Finnigan, Milan, Italy). We assumed that SOC was equal to the total carbon since there were no carbonates in our soil.

### 2.4. Data analysis

The mean respiration of the four core samples for each treatment was calculated for each measurement day. These means were then fitted to separate (one for each treatment) decaying exponential models as Equation (1) using R software (Oakland, CA, USA).

(1) $$R=a+b*\exp\left(-\frac{t}{c}\right)$$

where R is respiration (pmol·g^−1^·s^−1^), a is background or steady state respiration (pmol·g^−1^·s^−1^), b is initial minus background respiration (pmol·g^−1^·s^−1^), t is incubation time (days) and c is time constant (days).

We used one way and Repeated Measures Analysis of Variance (ANOVA) to test the effects of time and earthworms on soil respiration and soil SOC, DOC, MBC contents. We performed the least significant difference (LSD) test to compare the means of total CO_2_ emission, SOC, DOC and MBC for the different treatments. All statistical analyses were done by using SPSS 16.0 statistical software (IBM, Chicago, IL, USA).

## 3. Results

### 3.1. Soil Respiration under Different Treatments

**Figure 1:**
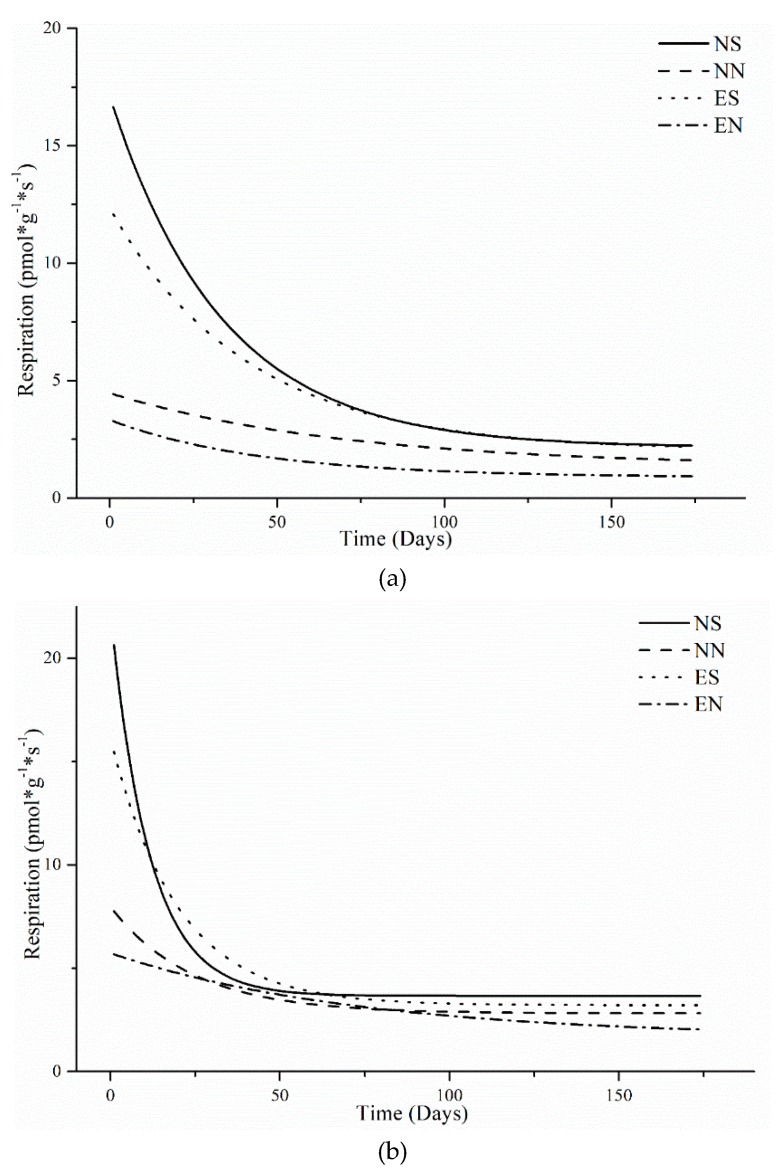
Soil respiration regression model over time under different treatments in CT (**a**) and NT (**b**).

The parameters of the respiration regression models (Equation (1)) are shown in Table 1. Both NS and ES had a strong initial effect under both CT and NT, but NN and EN had a much weaker initial starting value. Repeated measures anova showed soil respiration for all treatments significantly (*p* < 0.001) decreased with time in both CT and NT (Table 2), this decrease is also evident in Figure 1.

**Figure 2:**
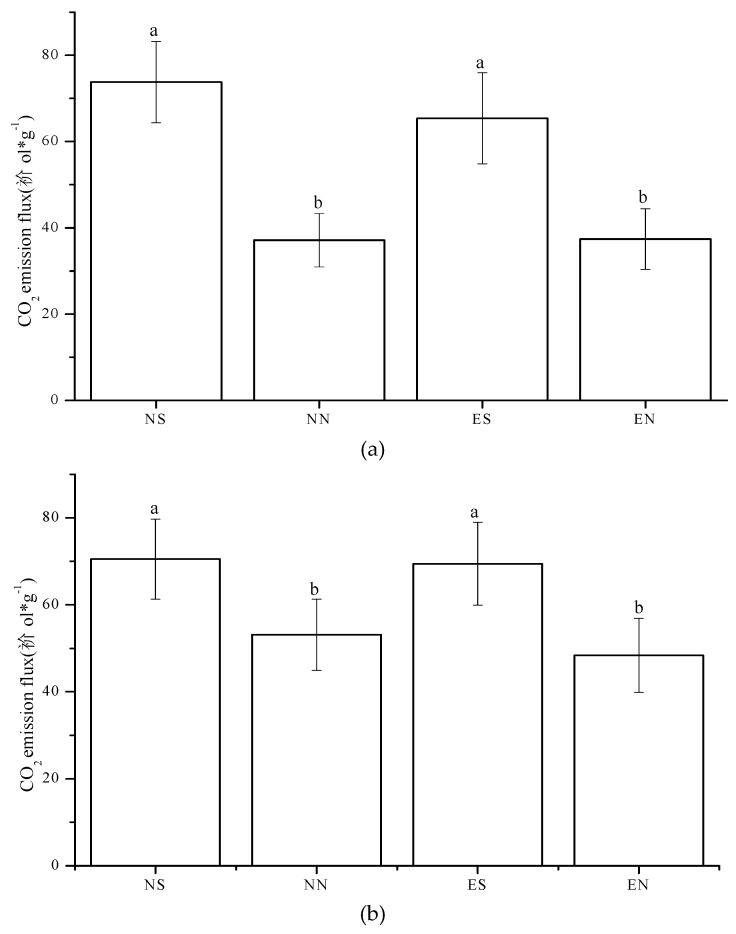
Total CO_2_ emission flux under different treatments for the duration of the experiment in CT (**a**) and NT (**b**) (mean value (standard error); Treatments indicated by the same letter are not significantly different at *p* < 0.05 on the LSD).

The total CO_2_ emission for each replicate calculated by numerical integration of the measured respiration rates over the 180 incubation period was higher in NS and ES than in NN and EN in both CT and NT (Figure 2).

### 3.2. The Impact of Earthworm and Residue on Total SOC

**Figure 3:**
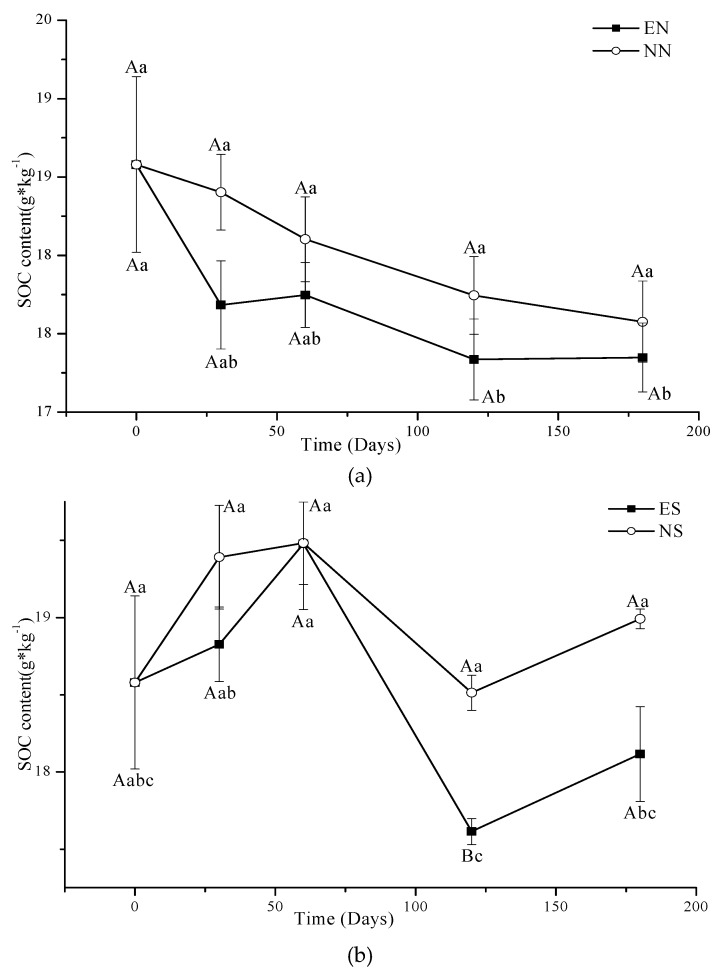
Concentrations of SOC in EN and NN (**a**), ES and NS (**b**) under different treatments in CT. (mean value ± standard error; Treatments indicated by the same upper case letter are not significantly different at *p* < 0.05 on the basis of one-way ANOVA in the same days; Days in the same treatment and indicated by the same lower case letter are not significantly different at P<0.05 on the basis of one-way ANOVA).

Under CT, the Anova showed that the effect of time on total SOC content was not significant in NN and EN (Table 2), but there was a general trend for a decrease over 180 days in NN. Post hoc pair wise analysis showed that the last 60 days of EN had significantly lower SOC than the initial value (*p* < 0.05) (Figure 3a). SOC content of NS and ES had an increasing trend over the first 60 days, and then significantly decreased in ES, and marginally decreased in NS under CT (Figure 3b).

**Figure 4:**
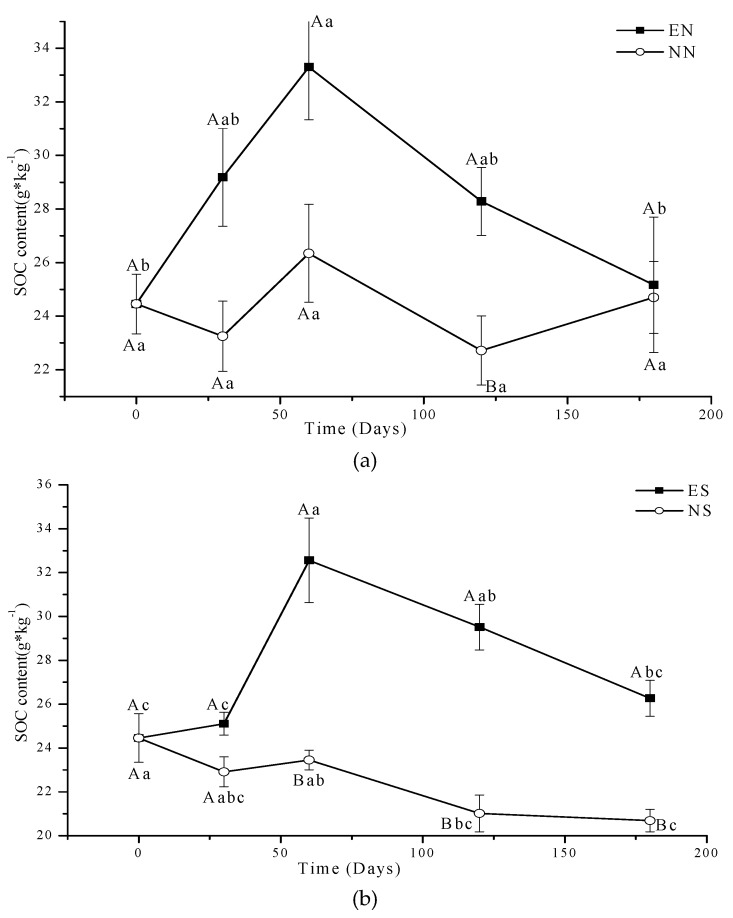
Concentrations of SOC in EN and NN (**a**), ES and NS (**b**) under different treatments in NT. (mean value ± standard error; Treatments indicated by the same upper case letter are not significantly different at *p* < 0.05 on the basis of one-way ANOVA in the same days; Days in the same treatment and indicated by the same lower case letter are not significantly different at P<0.05 on the basis of one-way ANOVA).

Under NT, SOC content of EN significantly increased in the first 60 days and then decreased, while NN remained stable during the 180 day incubation period (Fig. 4a). There were significant differences in SOC content in NS and ES with time (*p* < 0.05) (Table 2). SOC of ES remained stable for the first 30 days, significantly increased to 60 days and then decreased for the duration of the 180 day experiment. SOC content of NS significantly decreased with time under NT (Figure 4b).

### 3.3. The Impact of Earthworm and Residue on Active SOC Fractions

**Figure 5:**
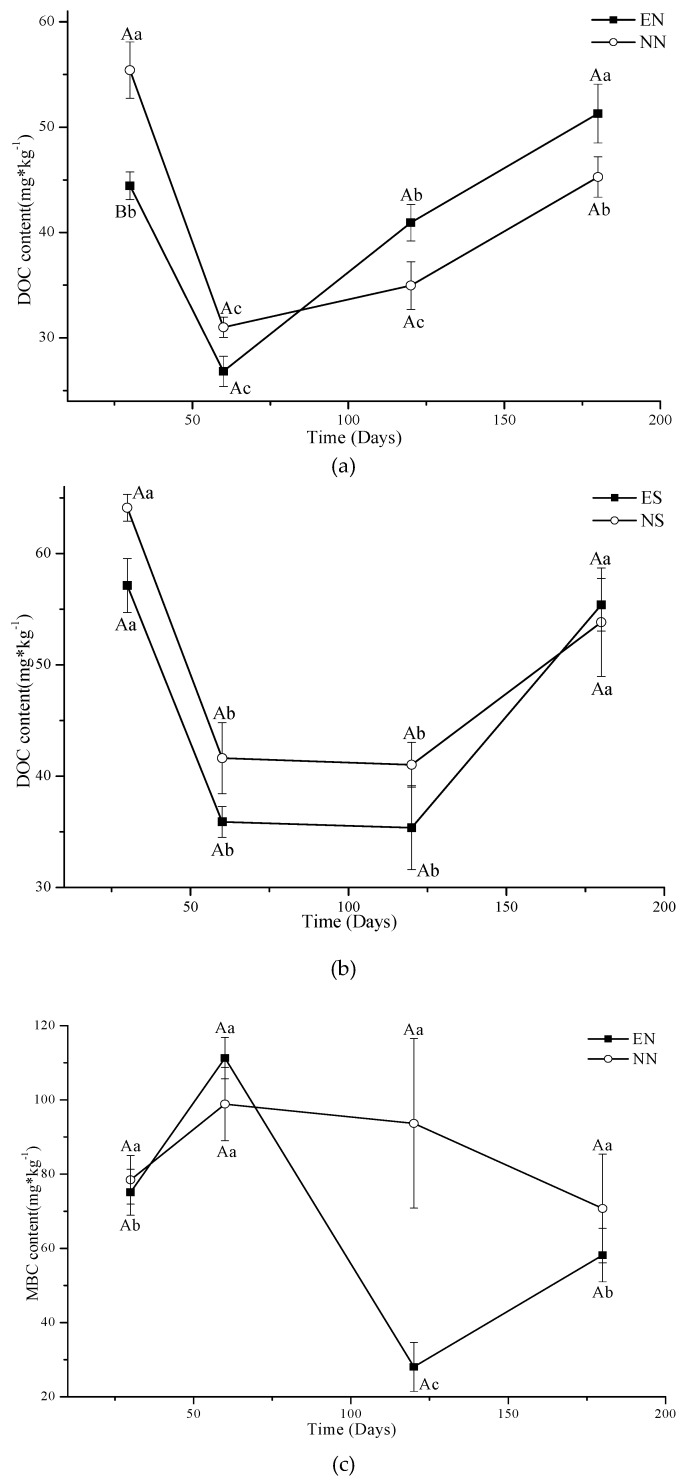

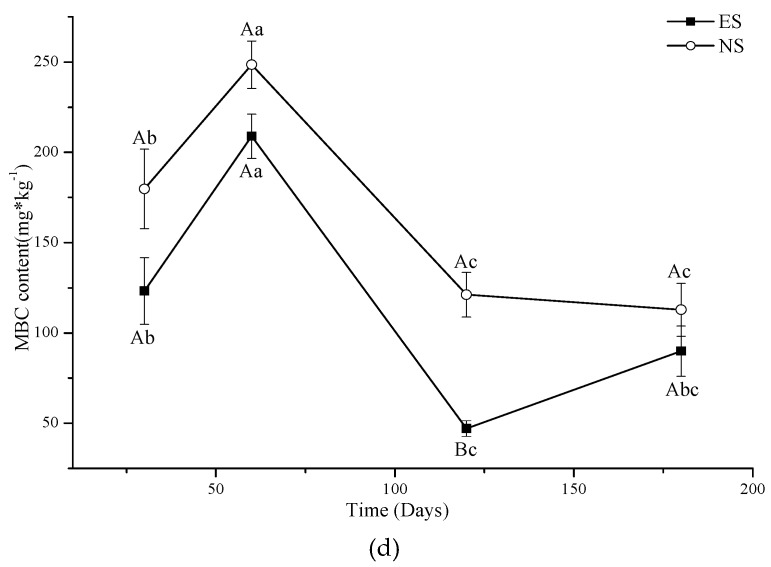
Concentrations of DOC in EN and NN (**a**), ES and NS (**b**) and MBC in EN and NN (**c**), ES and NS (**d**) under different treatments in CT. (mean value ± standard error; Treatments indicated by the same upper case letter are not significantly different at *p* < 0.05 on the basis of one-way ANOVA in the same days; Days in the same treatment and indicated by the same lower case letter are not significantly different at *p* < 0.05 on the basis of one-way ANOVA).

In CT, the DOC of all earthworm and residue treatments differed among different days (*p* < 0.05) (Figure 5a,b, Table 2). There was a general trend of an initial decrease in DOC content of all treatments from the starting date to 60 to 120 days, and then an increase in the later part of the experiments. There was a general trend for the DOC of both earthworm treatments (EN and ES) to approach the respective non-earthworm treatments (NN and NS) with time; the rate of change in EN was faster than that of NN. However, the only significant difference in DOC among the treatments was between EN and NN at the starting date. Time had a significant (*p* < 0.05) effect on MBC content (Table 2). MBC content for all treatments had an opposite trend to DOC in CT with a significant general initial increase followed by a decrease (Figure 5c,d).

An exception was the MBC of NN which remained relatively stable over time (Figure 5c). The difference in both DOC and MBC content of NS and ES gradually diminished over time.

**Figure 6:**
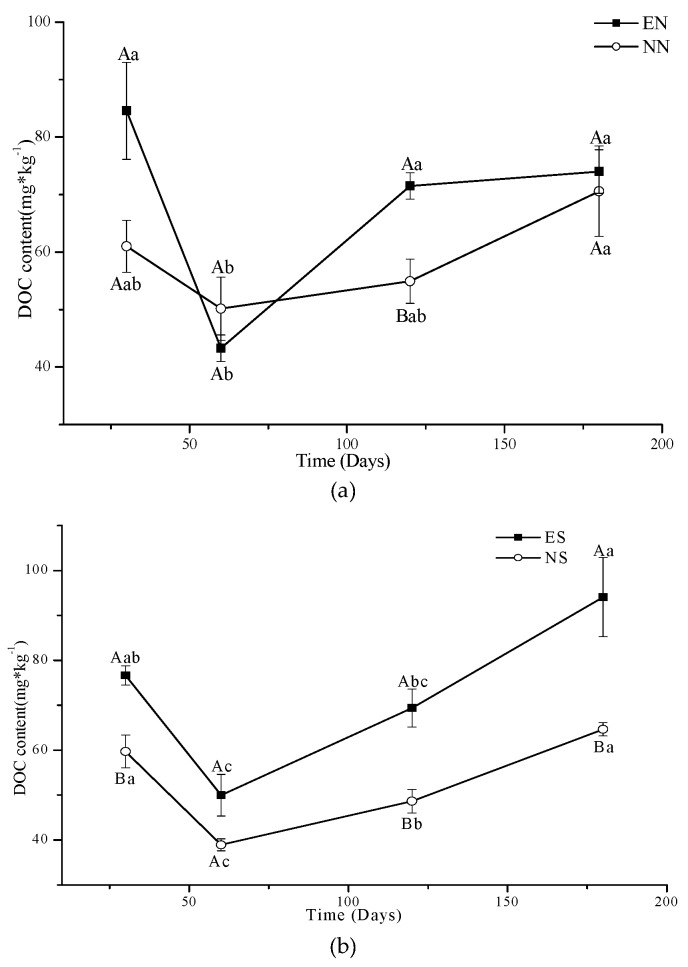

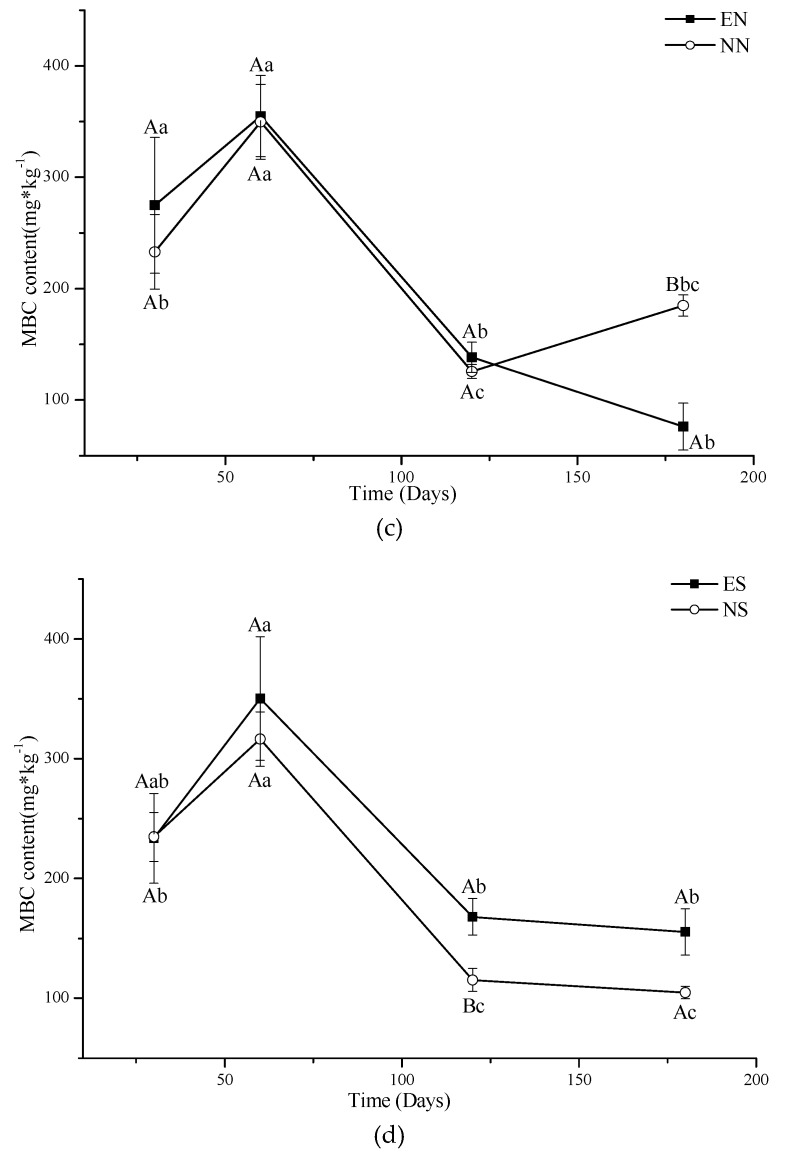
Concentrations of DOC in EN and NN (**a**), ES and NS (**b**) and MBC in EN and NN (**c**), ES and NS (**d**) under different treatments in NT. (mean value ± standard error; Treatments indicated by the same upper case letter are not significantly different at *p* < 0.05 on the basis of one-way ANOVA in the same days; Days in the same treatment and indicated by the same lower case letter are not significantly different at P<0.05 on the basis of one-way ANOVA).

In NT, the DOC content for all treatments had a pattern similar to the respective treatments in CT with an initial high value, a decrease at 60 days, and then an increase in the later part of the measurement period (Figure 5 and Figure 6). Time had a significant effect on DOC in NT with the exception of NN where DOC remained relatively stable during the 180 day incubation period (Figure 6a, Table 2). The DOC of NS and ES differed among different days with a decline in the first 60 days and an increase afterwards (*p* < 0.05). The difference in DOC content between NS and ES gradually increased with time (Figure 6b). Time had a significant (*p* < 0.05) effect on MBC for all treatments (Table 2). For all treatments, there was an initial increase in MBC in the first 60 days, and then a decrease at later times (Figure 6c,d); the trend was opposite to that for DOC. Similar to DOC, the difference in MBC content between NS and ES gradually increased with time (Figure 6d).

## 4. Discussion

### 4.1. The Effect of Earthworms and Residue on Soil Respiration

Numerous studies have confirmed that earthworms affect soil CO_2_ [22,33,34,35,36], through their direct and indirect impacts on the soil environment which depend on the quality of resources and microbial processes [22,37,38,39]. Earthworms enhance CO_2_ emissions [13,23,38,40]. Generally earthworm-induced CO_2_ emissions decreased over the duration of the experiment, and ceased to be significant beyond 200 days [22]. Our results showed that earthworms did not affect the soil respiration, while residue significantly increased the soil respiration both in CT and NT. Soil respiration of NT was greater than that of CT when residue was limited in NN and EN; NN and EN in NT had some surface residue from previous years. Zhang [23] stated that we have overestimated the CO_2_ emission by earthworms; however, earthworm gut and fresh earthworm casts may stimulate methanogenic activity [41], so the earthworm would increase CH_4_ emissions [42] by consuming the residue. The net CH_4_ production caused by earthworms is site-specific, and depends on soil moisture conditions and endemic methanogenic or methanotrophic bacteria [42,43,44]. It is likely that the net CO_2_ emission is also site-specific for similar reasons.

### 4.2. The Impact of Earthworm on SOC without and with Residue Returned

For CT with periodic soil disturbance to simulate cultivation, the respiration of microbes consumed some SOC, so the SOC content at 180 days was lower than that of 0 days in NN. Earthworm activity increased the soil organic matter (SOM) turnover (carbon and nitrogen mineralization) over time in agricultural soils creating favorable conditions for nutrient cycling [29]. Earthworms stimulate and accelerate SOM decomposition by enhancing microbial respiration [18,19], which explains why the SOC content was smaller in EN than that in NN at 30 days. Earthworms may reduce the pool size of potentially mineralizable C (PMC) and increase the pool sizes of both readily mineralizable C and stabilized C [23]; with a limited supply of SOC that is easy to digest by earthworms, the changes of SOC over time were small in EN of CT. Fahey et al. [9] suggested that earthworm invasions have the potential to reduce soil C storage in the upper 20 cm of the soil by 37%, echoing the results of Bohlen et al., [45] who found a 28% reduction in soil C in the upper 12 cm of a temperate hardwood forest. The new added residue and periodic stirring in NS and ES of CT would have a “priming effect” [46] with low initial total carbon content [47]. The residue would decompose quickly and marginally increase the SOC in the short term; following decomposition, the normal microbial activity would consume and decrease SOC resulting in the peak SOC observed in Figure 3b part way through the experiment.

Some studies have shown that SOC and MBC have a significant positive correlation [48,49]. In our CT soil with both earthworms and residue added, the SOC showed the same trend as MBC. Vineela et al. [49] showed that MBC also had a positive correlation with the microbe population and consequently, MBC can be a surrogate to represent the microbe population. The earthworms would initially use some SOC but the microorganisms need some time to decompose the new added residue into SOC which leads to a slight initial reduction in SOC; then the microorganisms would flourish with the abundant decomposed residue. Wang et al. [50] also found competition among the microorganisms; after the food resource is depleted, whereby they will compete and populations will decrease. As a result, the MBC changed with time [51,52].

There was a lot of native residue from previous years on the surface and distributed (roots) within the NT cores providing food for microorganisms and earthworms. We did not disturb the NT soil during the experiment, so the SOC content of NN did not change throughout the experiment in NT. Typically, earthworm presence is stimulated in NT systems with surface residue retention, where soil disturbance is minimal and food supply is relatively constant [53,54]. The earthworms prefer to consume the surface residue in NT instead of the SOC, and redistribute C via their casts and thus would increase the SOC in a short time and significantly enhance soil C stabilization [23]. At a later time, when surface residue is depleted, the earthworms would go down into the soil and then use the SOC of NT soil, and decrease the SOC of EN near the end of the experiment (still greater than NN) [13]. Since we added the new residue on the soil surface and did not disturb it with cultivation in ES and NS of NT, the soil microorganisms would use the new residue. Soil invertebrate fauna and microbes interact in the regulation of soil carbon (C) cycling processes, thereby affecting SOC dynamics and CO_2_ emissions [18,19]. In the NT soil with residue added, the new added residue would inhibit the residue decomposition by changing the community of microorganism or competition between the microorganism with limited N [55,56,57], so the SOC decreased with the time in NS (Figure 4b). However the increase in decomposition by earthworms was greater than inhibition of residue decomposition by microbes, which causes the MBC and SOC increase a little in the first 60 days, and then earthworms would go down into the soil, and consume the SOC, thereby decreasing the SOC; the SOC content of ES was still greater than that of NS.

### 4.3. The Impact of Earthworm on Active Fractions in the Soil

Perelo and Munch [58] suggested that microorganisms can use the DOC to increase their biomass. We disturbed the soil of CT to simulate cultivation by stirring at 30, 60, 120 and 180 days. The microorganisms would use DOC as an energy source and with time, increase their number and biomass. With more and more microorganisms, the competition with each other increases and causes the MBC to decrease and DOC to increase. This was illustrated by the negative correlation between MBC and DOC in our study which was similar to Yu et al. [59], but opposite to some other studies which showed that MBC had a positive correlation with DOC [60,61,62].

The earthworm gut and associated structures (casts, burrows, middens) form microhabitats that can support distinct microbial communities and greater microbial activity than the bulk soil [37,63,64], so the earthworms would stimulate microbial activity. The environmental condition for soil biota was better in NT than CT soil, because NT soil could increase the availability of soil organic matter and maintain less fluctuation in soil moisture and temperature [65,66]. The abundance and activity of soil biota in NT soil was higher than that in CT soil [32,67,68,69,70]. Nematode diversity and ratio of fungi:bacteria (F/B) is also affected by tillage [32]. Different tillage systems might result in different soil biological communities that promote or inhibit C storage [70]. Frouz et al., [71] also showed that earthworm had different effect on different soils because of different initial composition of C pools and perhaps also because of interaction between pools.

Prior to sampling for the incubation experiment, the CT soil was subjected to annual moldboard ploughing for a very long time, which is known to adversely affect some of the microorganisms; those that are left are better adapted and can tolerate changes imposed by periodic stirring in the CT soil, so the MBC of NN did not change during the time. There was a large diversity of microorganisms in NT, but many of microorganisms in NT soil were poorly adapted to soil disturbance; the microorganisms introduced by earthworm are also likely poorly adapted to soil disturbance similar to NT. So the microorganisms introduced by the earthworms must coexist and compete with the native microorganisms in the CT soil which has been subjected to cultivation.

The interaction of soil invertebrate fauna and microbes affects SOC dynamics [18,19]. Initially, the microorganisms that are decomposing the new residue and native microorganisms will compete with the microorganisms introduced by earthworms and result in lower MBC and DOC than without earthworms. With the cultivation in CT, the microorganisms introduced by residue and earthworms and native microorganisms must coexist, resulting in the difference in content of DOC and MBC between NS and ES gradually decreasing with time.

There was a lot of residue for earthworms and microorganisms to eat in the NT soil, so the DOC of NN showed no change over time and MBC fluctuated over time as the microorganisms competed with each other; when there were too many, the population decreased, and when the competition disappeared, they prospered again [50]. Microorganisms introduced by earthworms were not able to compete with native microorganisms in NT soil. The earthworms were stimulated in NT [53,54] and earthworms stimulate heterotrophic activity, strongly affecting decomposition processes through interactions with microbes, and macro- and microfauna [72,73]. Since we added the new residue in the NT soil and did not disturb it with cultivation in ES and NS of NT, the earthworm would use the new residue, and result in higher content of DOC and MBC in ES compared with NS. The microbes would consume the DOC and increase their abundance and competition with the final result that MBC would then decrease and DOC would increase [58]. Similarly, microorganisms introduced by earthworms and native microorganisms in NT would compete with the microorganisms introduced by the residue, leading to an increase in gaps in both DOC and MBC between NS and ES with time until all of the added residue became stabilized OM.

## 5. Conclusions

(1) Earthworms did not affect the emission of CO_2_, while residue significantly increased the emissions of CO_2_ in both CT and NT.

(2) The microorganisms use DOC to produce their microbial biomass, so DOC and MBC showed opposite trends in changes over the whole incubation period. The earthworms hastened this action in CT without residue return.

(3) The effect of earthworms on DOC and MBC gradually diminished with time in CT. The effect of earthworms on DOC and MBC increased with time in NT with residue addition.

(4) Earthworms hastened the SOC mineralization during the first 30 days, but the effect was lower at later times in CT. The newly added residue decomposed quickly to produce the SOC in 60 days and then decomposition slowed down in CT. The effect of earthworms and residue was combined into a single effect in CT.

(5) Earthworms decomposed native residue to increase the SOC in the first 60 days and then consumed SOC resulting in a decrease in SOC after 60 days in NT. The addition of new residue would inhibit the original residue decomposition but the effect was gradually weakened with time, and the acceleration of decomposition by earthworms was greater than inhibition of new residue decomposition by microbes. The effect of earthworms and residue was combined into a single effect in NT. Earthworms enhanced mineralization of SOC in CT but generated SOC in NT. Further study is needed to understand how soil microbes and earthworms interact in SOC dynamics.

## Figures and Tables

**Table 1:** The coefficients of respiration regression model (respiration = a + b*exp(−t/c)) for the different earthworm and residue treatments in CT and NT. Standard errors of the coefficients are shown in parenthesis.

Tillage	Coefficient	NS	NN	ES	EN
CT	a (pmol·g^−1^·s^−1^)	2.15 (0.74)	1.34 (1.09)	2.03 (0.46)	0.88 (0.31)
	b (pmol·g^−1^·s^−1^)	14.94 (1.03)	3.13 (0.94)	10.30 (0.52)	2.45 (0.33)
	c (days)	33.45 (6.48)	71.02 (61.44)	40.91 (6.42)	45.07 (19.28)
NT	a (pmol·g^−1^·s^−1^)	3.66 (0.36)	2.82 (0.23)	3.21 (0.42)	1.65 (0.92)
	b (pmol·g^−1^·s^−1^)	18.50 (1.22)	5.15 (0.44)	12.89 (0.95)	4.08 (0.79)
	c (days)	11.57 (1.27)	24.19 (4.89)	19.90 (3.19)	73.75 (40.30)

**Table 2:** The P values of respiration (R), SOC, DOC and MBC and changes with time for the different earthworm and residue treatments in CT and NT.

Treatment	CT				NT			
	R	SOC	DOC	MBC	R	SOC	DOC	MBC
NS	0.000	0.196	0.002	0.001	0.000	0.034	0.000	0.000
NN	0.000	0.313	0.000	0.636	0.000	0.571	0.195	0.001
ES	0.000	0.039	0.000	0.000	0.000	0.005	0.003	0.017
EN	0.000	0.191	0.000	0.000	0.000	0.084	0.002	0.003
